# *Notes from the Field:* Impact of a Mass Drug Administration Campaign Using a Novel Three-Drug Regimen on Lymphatic Filariasis Antigenemia — American Samoa, 2019

**DOI:** 10.15585/mmwr.mm6921a3

**Published:** 2020-05-29

**Authors:** Marisa A. Hast, Aifili Tufa, Tara A. Brant, Lynette Suiaunoa-Scanlan, Janet Camacho, June Vaifanua-Leo, Keri Robinson, Emily Dodd, Ben Sili, Loretta S. Lees, Kimberly Y. Won, Fara Utu

**Affiliations:** ^1^Epidemic Intelligence Service, CDC; ^2^Division of Parasitic Diseases and Malaria, Center for Global Health, CDC; ^3^American Samoa Department of Health; ^4^Pacific Island Health Officers’ Association, American Samoa.

Lymphatic filariasis is a debilitating and disfiguring mosquitoborne parasitic disease. As part of the Global Programme to Eliminate Lymphatic Filariasis, the World Health Organization (WHO) recommends at least five rounds of annual mass drug administration (MDA) in areas with endemic disease to reduce incidence and prevalence (*1*). Onward transmission is expected to end once community prevalence falls below 1% (*1*).

American Samoa, located in the southern Pacific Ocean, is the only U.S. territory with evidence of ongoing lymphatic filariasis transmission. After 7 years of MDA (2000–2006), the prevalence of lymphatic filariasis antigenemia in American Samoa declined from 16.5% to 2.3%, and MDA was stopped (*2*,*3*). In 2016, a household survey among 2,507 participants revealed that the prevalence of antigenemia had rebounded to 6.2%, and transmission was ascertained to be widespread across the territory (*4*). MDA was resumed in 2018 using a novel three-drug regimen of ivermectin, diethylcarbamazine, and albendazole, which has been shown to more effectively clear filarial larvae from the blood than the standard two-drug treatment of albendazole with diethylcarbamazine or ivermectin alone (*5*,*6*). This WHO-recommended three-drug regimen is anticipated to accelerate progress toward global elimination goals in areas without other filarial infections that would contraindicate the use of diethylcarbamazine (onchocerciasis) or ivermectin (loiasis).

During July 11–August 17, 2019, the American Samoa Department of Health (ASDOH) conducted a survey in collaboration with CDC and the Pacific Island Health Officers’ Association* to determine the effect of three-drug MDA on lymphatic filariasis prevalence in American Samoa. Households were selected from all 68 villages on the main islands of Tutuila and Aunu’u using systematic random sampling. Children aged 5–9 years and villages previously known to have high transmission rates were oversampled. Eligible household members aged ≥5 years with provision of informed consent were administered a questionnaire and provided a blood specimen for lymphatic filariasis antigen testing using the Alere filariasis test strip (Abbott). All participants who received antigen-positive test results were offered treatment.

ASDOH visited 1,865 households and enrolled 2,081 persons in the survey. A total of 47 participants with a positive antigen test for lymphatic filariasis were identified. Cases were geographically dispersed; however, a large proportion of cases were found along the western coast of Tutuila. By age, the antigen test positivity rate was 1.1% among children aged 5–9 years and 2.9% among household members aged ≥10 years. After adjusting for age and location, the overall prevalence of lymphatic filariasis antigenemia in American Samoa was estimated to be 2.7%. Adjusted prevalence of antigenemia was higher among males (4.8%) than among females (1.0%) (p<0.001), and this pattern was consistent across age groups (Figure). Differences in antigen prevalence by sex can be attributed in part to differences in MDA participation. Nonparticipation in the 2018 MDA was 35.2% among participants with positive antigen test results, compared with 22.2% among participants with negative antigen test results, and antigen prevalence among men aged >40 years who did not participate in the MDA was >10%.

**FIGURE:**
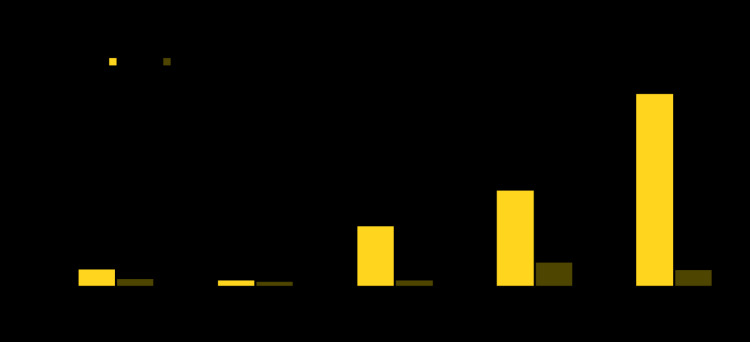
Prevalence of lymphatic filariasis antigenemia following mass drug administration using a novel three-drug* regimen, by age group and gender — American Samoa, 2019 * Ivermectin, diethylcarbamazine, and albendazole.

These results indicate that lymphatic filariasis antigenemia has declined since 2016 but remains above the 1% WHO threshold in all age groups, suggesting that lymphatic filariasis transmission in American Samoa is ongoing. To interrupt transmission in this setting, American Samoa should consider following WHO recommendations (*5*) and continue annual three-drug MDA with appropriate monitoring of progress toward elimination until targets are met. Lymphatic filariasis control activities should target high-prevalence sectors of the population, including adult men, to ensure that this population is adequately covered in the future.

## Footnotes

* https://www.pihoa.org/.
